# Impact of mass drug administration with ivermectin, diethylcarbamazine, and albendazole for lymphatic filariasis on hookworm and *Strongyloides stercoralis* infections in Papua New Guinea

**DOI:** 10.1371/journal.pntd.0012851

**Published:** 2025-03-10

**Authors:** Jannet A. Tobon Ramos, Tobias Maure, Lenore Carias, Daphne Lew, Charles Goss, Anna Samuel, Livingstone Tavul, Peter U. Fischer, Gary J. Weil, Moses Laman, Leanne J. Robinson, William Pomat, Christopher L. King

**Affiliations:** 1 Department of Pathology, Center for Global Health and Diseases, Case Western Reserve University, Cleveland, Ohio, United States of America; 2 Department of Infection and Immunity, Papua New Guinea Institute of Medical Research, Goroka, Eastern Highlands Province, Papua New Guinea; 3 Institute for Informatics, Data Science, and Biostatistics, Washington University School of Medicine, St. Louis, Missouri, United States of America; 4 Department of Medicine, Division of Infectious Diseases, Washington University School of Medicine, St. Louis, Missouri, United States of America; 5 Department of Health Science and Epidemiology, Burnet Institute, Melbourne, Victoria, Australia; 6 Medical Services, Veterans Affairs Medical Center, Cleveland, Ohio, United States of America; University of Agricultural Sciences and Veterinary Medicine Cluj-Napoca, Life Science Institute, ROMANIA

## Abstract

**Background:**

Persons with lymphatic filariasis (LF) are often co-infected with soil-transmitted helminths. A single co-administered dose of ivermectin/diethylcarbamazine/albendazole (IDA) is recommended by WHO for mass drug administration (MDA) for LF instead of diethylcarbamazine/albendazole (DA) in Papua New Guinea (PNG). We compared the effectiveness of a single round of MDA with IDA or DA on hookworm and strongyloidiasis in PNG.

**Methodology/Principal Findings:**

This study was conducted as part of a cluster randomized trial of MDA with IDA versus DA for LF in individuals willing to provide stool and blood samples at baseline and 12 months after MDA. Participants from 23 villages were included in the clinical trial. Primary outcomes were changes in hookworm prevalence and infection intensity assessed by Kato Katz and *Strongyloides* prevalence by serology.

Hookworm prevalence at baseline was 78% (91/117) and 80% (119/149) in villages assigned to DA and IDA treatment, respectively. Twelve months post-MDA, hookworm prevalence decreased to 56.5% in DA- and 34.4% in IDA-treated villages, respectively (*p*<0.001, both comparisons). The proportion of individuals with moderate to heavy infection (>2000 egg per gram (EPG)) similarly decreased from 8.7% to 1.5% after DA (*p* = 0.001) and from 5.7% to 1.0% after IDA (*p* = 0.002). Using a logistic regression model adjusting for age, gender, baseline hookworm prevalence, and village drug coverage, IDA resulted in a 45% greater reduction in hookworm prevalence than DA (Odds ratio 0.55, 95% CI [0.31,0.99], *p* = 0.049). MDA also reduced hookworm transmission. *Strongyloides* seroprevalence at baseline was 68% (192/283) and 62% (180/290) in IDA and DA villages, respectively, with 49% becoming seronegative in the IDA versus 23% in DA villages at 12 months (*p* = 0.0001).

**Conclusions/Significance:**

MDA with IDA was more effective than DA for reducing hookworm and *Strongyloides* infections in PNG, extending the benefit of MDA with IDA beyond its effect on LF.

## Author summary

The study examined the benefit of ivermectin/diethylcarbamazine/albendazole (IDA) compared to diethylcarbamazine/albendazole (DA) on soil-transmitted helminths and *Strongyloides* worms as part of a mass drug administration (MDA) campaign for lymphatic filariasis in Papua New Guinea. Villages were randomized to receive IDA or DA, and all eligible community members received one or the other treatment regimen. At baseline, overall hookworm infection was present in 78% of individuals on stool examination using the Kato Katz assay, and 62-68% were serologically positive for *Strongyloides* in both treatment arms. One year after a single round of MDA, residents of villages receiving IDA had a 45% greater reduction in hookworm prevalence versus DA, after adjusting for potential influencing factors. Notable was a 47% reduction in hookworm prevalence and an 89% reduction in the intensity of infection among untreated individuals surveyed 12 months after MDA in IDA-treated communities, much greater than that observed in DA-treated villages. This shows a community effect of MDA on hookworm, suggesting a decrease in hookworm transmission. MDA reduced strongyloidiasis seroprevalence in IDA-treated villages by 49% 12 months later, but no significant change in seroprevalence was observed in DA-treated villages. This highlights IDA’s added public health benefit for controlling soil-transmitted helminth infections.

## Introduction

Soil-transmitted helminth infections (STH) are common parasitic infections in many low and middle-income countries. Hookworm infections caused by *Necator americanus* and *Ancylostoma duodenale* are prevalent in the Western Pacific region [1]. In Papua New Guinea (PNG), *N. americanus* is the predominant hookworm species, with an estimated prevalence of 60 – 100% [2–4], contributing to the high prevalence of iron deficiency anemia in the country [5]. Strongyloidiasis is also an STH, with biological similarities to hookworm. The free-living larvae of *Strongyloides stercoralis* and *N. americanus* directly penetrate the skin to establish infection. The warm, loamy soils common in coastal areas of PNG favor the survival of their free-living larvae and support a high prevalence of skin-penetrating STH in these areas where mosquito-borne lymphatic filariasis (LF) is co-endemic.

Globally, an estimated 440 million people were infected with hookworm in 2010 [6]. Although this number has decreased to an estimated 230 million in the last decade, more than 2.0 billion people remain at risk for the infection [7,8]. The prevalence of *S. stercoralis* in PNG and globally is not as well established because of the poor sensitivity of diagnostic techniques [9,10]. More sensitive molecular and serological assays suggest that the global burden of strongyloidiasis could be as high as 617 million [11,12].

The WHO recommends preventive chemotherapy for STH using benzimidazole medications (albendazole [ALB] or mebendazole) for pre- and school-aged children and reproductive-aged women who are at the highest risk of developing morbidity due to STH infections. Preventive chemotherapy for these high-risk populations has contributed to the global decline in hookworm prevalence [13]. However, older children and adults often have the heaviest burdens of hookworm and strongyloidiasis that can sustain transmission in communities if left untreated with this targeted strategy [14,15]. Mass drug administration programs for LF, including ALB, provide STH treatment for older children and adults, reducing STH’s burden in areas with MDA for LF elimination [16,17].

Benzimidazoles have limited efficacy against *Trichuris* *spp* and strongyloidiasis. Ivermectin (IVM) is the drug of choice for treating *S. stercoralis*. A recent Cochrane Review compared IVM versus ALB for treating *S. stercoralis.* It concluded that IVM is superior to ALB, with a cure rate of IVM estimated at 74% to 84% vs. ALB at 48% [18]. Combining IVM plus ALB is also more efficacious for treating *Trichuris spp* than either drug alone, although the efficacy of the combination therapy is variable in different countries [19–22]. Albendazole is otherwise highly effective against other STHs. Clinical trials assessing the efficacy of IVM plus ALB for *Trichuris* also reported the impact of IVM plus ALB on hookworm infection as a secondary outcome, and the combination was not clearly superior to ALB alone [19,21–23]. However, there is a paucity of studies evaluating the added benefit of IVM and ALB combination therapy as compared to ALB without ivermectin on hookworm and *Strongyloides* in the context of LF MDA campaigns.

The high prevalence of *S. stercoralis* in SE Asia is notable, but there is little access to ivermectin and limited or non-existent strategies for *Strongyloides* control [12,24]. The community-based studies on LF will help inform the value of MDA with ivermectin for possible *Strongyloides* control in SE Asia. This is timely as the WHO roadmap for NTDs 2021–2030 included *Strongyloides* as an STH target for control [25].

In 2017, WHO recommended triple-drug therapy (co-administered IVM, DEC, and ALB) for MDA in LF-endemic areas outside sub-Saharan Africa that have either not started MDA or have failed to interrupt transmission with multiple rounds of DEC plus ALB [26]. One hundred sixty-six million doses of IDA were distributed in 25 LF-endemic countries from 2018–2022 [27]. To better evaluate how IDA impacts other helminth infections in MDA campaigns for LF, we examined the effectiveness of MDA with IDA versus DA on hookworm and strongyloidiasis as a secondary outcome in a cluster randomized trial in PNG to test the effectiveness of IDA compared to the two-drug regimen of DEC plus ALB.

## Methods

### Ethics statement

Institutional review boards at University Hospitals Cleveland Medical Center (No. 12-17-23), the Papua New Guinea Institute of Medical Research (PNG IMR, No. 1716), and the Papua New Guinea Medical Research Advisory Committee (No. 17.48) approved the trial. All adult participants provided written informed consent. For participating children, assent from children 12 and older and written consent from at least one parent or guardian was obtained. The trial was registered at ClinicalTrials.gov (trial registration number NCT02899936 and NCT03352206).

### Study design and participants

This study was conducted as part of an open-label cluster randomized clinical trial to assess the safety and effectiveness of IDA versus DA for LF. We performed cross-sectional STH surveys to assess the impact of IDA versus DA on soil-transmitted helminths in communities [28]. This study was conducted in the Bogia district, Madang province, PNG, between 2016 and 2018. Participants from 24 villages were included in the clinical trial. Stool samples were collected from village residents willing to provide a specimen before MDA and 12 months after treatment. Stool collection depended on participants’ willingness to provide stool samples, which was more acceptable in some villages than others. No stool samples were collected in one DA-treated village because of a rumor of witchcraft. Blood samples using finger prick were collected from all the LF clinical trial participants, including those with stool samples. None of the participants had received previous MDA or treatment for LF or STH. Participants were eligible for inclusion if they were five years or older, had no evidence of severe chronic illness, were not pregnant, and were not allergic to the trial drugs.

### Outcomes

The primary outcomes assess the impact of IDA versus DA treatment for LF on non-targeted STH organisms: hookworm (assessed by Kato-Katz quantitative egg detection) and *Strongyloides spp* (assessed by antibody responses to NIE antigen) 12 months after MDA.

### Randomization and masking

Randomization was performed at the village level. Study statisticians identified pairs of villages with similar population sizes and LF infection rates as previously described [29] and randomly assigned (1:1 using SAS random number generator) MDA with IDA or DA for each pair. Randomization was performed before enrollment so community members and investigators administering drugs knew treatment allocation. Staff reading the slides for STH were masked to treatment allocation by using identification numbers that could not be linked to the village without a key held by the study statisticians.

Directly observed treatment included a single dose of the two-drug regimen of DEC (6 mg/kg body weight) plus ALB (400 mg fixed dose) or a single dose of the three-drug regimen of IVM (200 µg/kg body weight) plus DEC (6 mg/kg body weight) plus ALB (400 mg). Merck Sharp & Dohme (MSD), also known as Merck & Co., Inc. (Kenilworth, NJ, USA), donated IVM. The PNG Ministry of Health provided ALB (produced and donated by GlaxoSmithKline, GSK) and DEC (made and donated by Eisai Co.) from existing stocks. Treatment coverage varied with age: drugs were ingested by a mean of 35.1% of children aged 5-9 in the triple-drug regimen group compared with 40.2% in the two-drug regimen group, by 72.7% of adolescents (aged 10-17) in the triple-drug regimen compared with 75.7% in the two-drug regimen group, and by 71.8% of adults (aged 18 years and older) in the triple-drug regimen group compared with 69.8% in the two-drug regimen group [29].

### Field procedures

Before MDA, between 8 – 11 PM, finger prick blood samples were collected and applied to a Whatman 3MM filter paper strip (Whatman Inc, Florham Park, NJ), dried overnight, and stored in individual plastic bags with silica gel packets at -20˚C for subsequent serological evaluation of *S. stercoralis*. Dried blood spots (DBS) were only collected at baseline and 12 months follow-up surveys.

On the evening of the registration for the LF study, all individuals who agreed to provide stool samples were educated about the importance of intestinal parasites and the survey’s objectives. A labeled stool container was provided to each participant, and samples were collected the following morning on ice packs and immediately taken to the field laboratory for Kato Katz examination. The remaining sample was applied to Whatman FTA cards (Whatman, Inc.), dried, put in individual plastic bags with silica gel packets, and stored at room temperature for subsequent DNA extraction and real-time PCR evaluation for *S. stercoralis*.

The DBS samples were transported to PNG IMR in Madang, PNG, for storage and later transferred to Case Western Reserve University (CWRU) in Cleveland, Ohio, USA for *Strongyloides* serology. The Whatman FTA cards used for stool samples were transported to PNG IMR in Goroka for DNA extraction and PCR assays. The remaining extracted DNA was transferred to CWRU to validate real-time PCR results.

### Laboratory procedures

#### Kato Katz.

Fresh stool was evaluated in the field laboratory by the Kato Katz method described elsewhere [30]. The samples were prepared in duplicate and read by two independent readers within 30 – 60 minutes of preparation and 8 hours or less after collection on ice packs. The readers counted and recorded all types of parasitic eggs. The readers of the KK slides were trained with experienced microscopists from outside institutions before the study. The study PI and external investigators experienced with KK technique randomly checked slides for accuracy during the study.

### PCR for *Strongyloides* infection

Stool samples were analyzed by real-time quantitative PCR for *Strongyloides spp*. DNA was extracted from FTA cards using a QIAamp DNA stool mini kit (Qiagen, Hilden, Germany) with a modified protocol. In brief, two circles were cut from an FTA card, cut into little pieces, and put into a 1.7 ml microcentrifuge tube; then 1 ml of ASL stool lysis buffer (Qiagen) was added, vortex for 30 seconds and centrifuged at 13,000 rpm for 1 minute in a microfuge. Samples were stored overnight at 4˚C. The following day, samples were placed in a water bath at 95˚C for 15 minutes and centrifuged at 13,000 rpm for 10 minutes. The supernatant (~ 600 µl) was transferred into a new 1.7 ml microcentrifuge tube, then the manufacturer’s protocol was followed. DNA samples were stored at -20˚C until real-time PCR was performed. Because non-*S. stercoralis* species have been previously detected in humans in PNG [4,31,32]; we initially evaluated all the samples using a set of primers and a probe genus-specific for *Strongyloides spp* [33] that targets the 18s rRNA gene. Next, all positive samples for *Strongyloides spp* were assessed with a different set of primers and a species-specific probe for *S. stercoralis* [34] targeting high copy number non-coding repeat DNA sequences (Table 1). To quantify each parasite’s amplicon copy number, we created a standard curve using purified DNA from an *E. coli* plasmid for *S. stercoralis*. DNA was extracted from L3 *S. stercoralis* larvae provided by Dr. Peter Fischer’s laboratory at Washington University St. Louis.

**Table 1:** Primers and detection probes used for Real-Time PCR.

Parasite/ Target	Forward Primer	Reverse Primer	Probe
***Strongyloides sp*/ 18s rRNA gene**	5’ GAA TTC CAA GTA AAC GTA AGT CAT TAG C 3’	5’ TGC CTC TGG ATA TTG CTC AGT TC 3’	5’ -/56-HEX/ACA CAC CGG/ZEN/CCG TCG CTG C/3IABkFQ/-3’
***Strongyloides stercoralis*/ non-coding repeat DNA sequences**	5’- CGC TCC AGA ATT AGT TCC AGT T-3’	5’ GCA GCT TAG TCG AAA GCA TAG A -3’	5’ -/56-FAM/ACA GTC TCC/ZEN/AGT TCA CTC CAG AAG AGT/3IABkFQ/ -3’

3IABkFQ: Iowa Black Fluorescent Quencher

### Serology methods

Using the method of Corran et al. 2008 [35], assuming a 40% hematocrit, a 3.5 mm diameter punch from a dried blood spot using Whatman 3MM filter paper corresponds to 1.38 µl of plasma. To validate the ability to recover immunoglobulins from plasma versus DBS, we performed an ELISA to detect total IgG in plasma and a DBS sample from a volunteer. Plasma IgG was 8.32 mg/ml, whereas that extracted from the DBS was 7.78 mg/ml of IgG, equivalent to 93% of the total IgG found in the plasma sample. This analysis was repeated on two additional volunteers with similar findings.

Dried blood spots (DBS) were removed from -20˚C and allowed to return to ambient temperature. To elute sera from DBS, 3.5 mm discs were punched from the spots (4 discs per sample) with a leather puncher; they were placed into 2 ml deep 96-wells polypropylene plates and added 520 µl PBS-T 0.05% (130 µl per disc; 1:100 dilution of plasma). Plates were sealed with sealing film and placed overnight at ambient temperature on a rotary shaker at two rev/sec. The next day, plates were centrifuged, and the eluate was transferred to 1.5 ml microcentrifuge tubes and stored at 4˚C or -20˚ C for long-term storage before performing the ELISA.

The Strongy Detect IgG ELISA kit (InBios, Seattle, WA) is a sandwich-type ELISA that detects total IgG antibodies to *Strongyloides* recombinant immunodiagnostic antigen (NIE) in serum. The NIE antigen is a 31-kDa recombinant antigen derived from an *S. stercoralis* L3 cDNA library [36]. The manufacturer’s protocol was followed with minor modifications. Negative and positive control samples provided with the kit were diluted to 1:100. Next, DBS eluted samples were diluted to 1:4 (1:400 of plasma) in 96-well polypropylene microplates using dilution buffer (PBS-T 0.5% plus proclin-300). Then 100 µl of the diluted samples were added to 96 well microtiter plates pre-coated with SRA, sealed, and incubated at 37˚C for 30 minutes. After the incubation, wells were washed six times with an automatic plate washer (Biotek Elx50, Winooski, VT) using 1x wash buffer (PBS-T; 300µl each wash cycle), then 100 µl of 100X enzyme conjugate (mouse monoclonal anti-human IgG conjugated with horseradish peroxidase in Tris buffer, diluted in PBS buffer solution) was added, the plates were sealed, incubated at 37˚C for 30 minutes and washed six times. Finally, 100 µl of tetramethylbenzidine (TMB) substrate was added to wells and incubated at room temperature in a dark drawer for 10 minutes before adding 50 µl of stop solution (1N sulfuric acid). OD values were read at 450 nm using a VersaMax ELISA reader (Molecular Devices, San Jose, CA).

### Statistical analysis

Statistical analyses were performed using SPSS Statistics software version 25 (IBM, Armonk, NY), Prism software version 8.1.1 (GraphPad, San Diego, CA), and SAS version 9.4 (SAS Institute, Cary, NC). Categorical variables were compared with Fisher’s exact test (two-tailed). The comparison of continuous variables across groups was analyzed using the Mann-Whitney U test (two-tailed) because the data did not have a normal distribution. Chi-square tests were used to determine the difference in the prevalence of infection between groups. Wilcoxon matched-pairs sign test was used to analyze NIE ELISA pre- and post-treatment changes. Differences were considered statistically significant if the *P-value* was less than 0.05 for all analyses. Estimates predicting hookworm infection were generated using a logistic mixed model (PROC GLIMMIX in SAS v9.4) to compare changes in hookworm prevalence across time and between treatment arms, adjusting for age, sex, village coverage at baseline, and baseline hookworm prevalence. The model included the follow-up time points of 12 months, treatment arms, and interaction between treatment and time as fixed effects.

The receiver operating characteristic curve (ROC) used to calculate NIE ELISA assay sensitivity, specificity, and the optimal cut-off was calculated using data from DBS samples of *S. stercoralis*-positive individuals (based on stool PCR), positive control samples provided with the kit; negative DBS samples from North American controls, and negative control samples supplied with the kit.

## Results

### Study population characteristics

**Fig 1:**
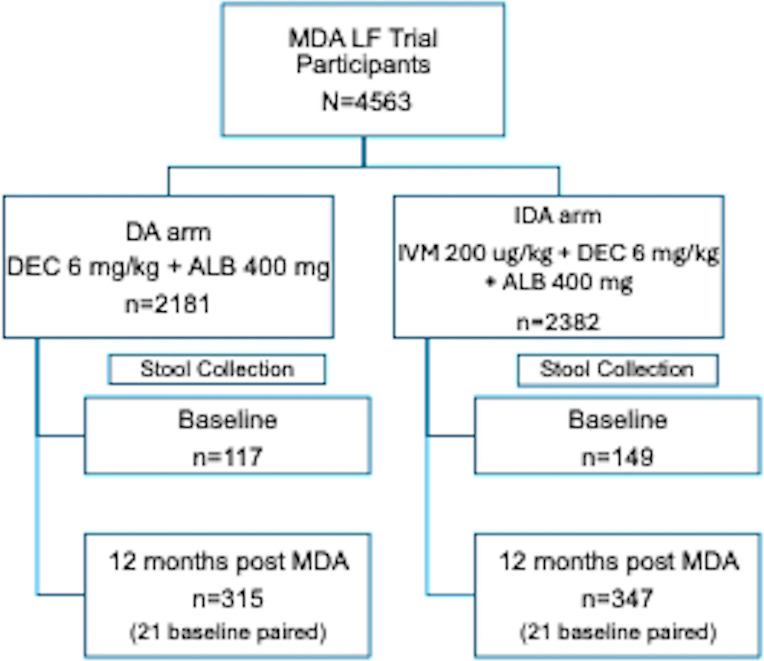
Study Profile. The number of individuals providing stool samples in each treatment arm is shown at baseline and 12 months post-MDA. Twenty-one individuals in both treatment arms had paired samples at baseline and 12 months follow-up. Stool samples collected at baseline and at 12 months were from 12 of 12 clusters in the IDA treatment arm and 11 of 12 clusters in the DA arm.

In total, 4563 individuals participated in the LF cluster-randomized trial: 2181 in the DA arm (*N* = 12 villages) and 2382 in the IDA arm (*N* = 12 villages, Fig 1). Individuals were asked to provide a stool sample whether or not they received MDA. Only a subset of participants agreed to provide stool samples from the different clusters; 266 participants at baseline (Table 2) and 650 individuals at 12 months (42 also had baseline samples, Table 2). Their demographic data and sample size are shown in Table 2 for each treatment arm.

**Table 2:** Hookworm prevalence and intensity of infection by Kato Katz at baseline (Panel A) and 12 months (Panel B) after treatment.

Treatment Arm	*n*	Median Age, Years (range)	Gender Male %	No. Positive (% Positive; 95%CI)	Geomean EPG^*^ (95% CI)	Arithmetic mean EPG (95% CI)
**Panel A. Baseline**						
**DA**	117	27 (6–68)	60	91 (78; 69, 85)	81 (50, 134)	527 (371, 682)
**IDA**	149	22 (5–69)	56	119 (80; 73, 86)	58 (39, 86)	305 (216, 394)
*P-*value		0.01	0.57	0.88	0.08	0.08
^*^Eggs Per Gram.						
**Panel B. 12 months**						
**Treatment Arm**	***n***	**Median Age, Years (range)**	**Gender Male %**	**No. Positive (%Positive; 95%CI)**	**Geomean EPG (95% CI)**	**Arithmetic mean EPG (95% CI)**
**DA**	310	18 (5–71)	55	175 (57; 51, 62)	12 (10,17)	133 (95, 171)
**IDA**	340	21 (5–77)	52	117 (34; 29,40)	4 (4,7)	173 (76, 271)
*P-*value		0.30	0.34	<0.001	<0.001	0.46

* Egg reduction rate relative to baseline (pre-treatment).

At baseline, stool examination by Kato Katz revealed mostly hookworm ova, rare Ascaris eggs (N = 2) and rare *Strongyloides* larvae (*N* = 2, later confirmed by PCR). At baseline, hookworm prevalence was comparable between the two arms (78 and 80%), with a trend toward heavier infection in the DA treatment villages (Table 2A). PCR of the stool samples confirmed that all hookworm infections were *N. americanus.*

Before treatment, hookworm prevalence and intensity were similar between sexes; females, 87 of 112 (78%) positive, geomean=69 ± 13 *SD* eggs per gram (epg), and males, 121 of 154 (79%) positive, geomean=66 ±11 *SD* epg.

**Fig 2:**
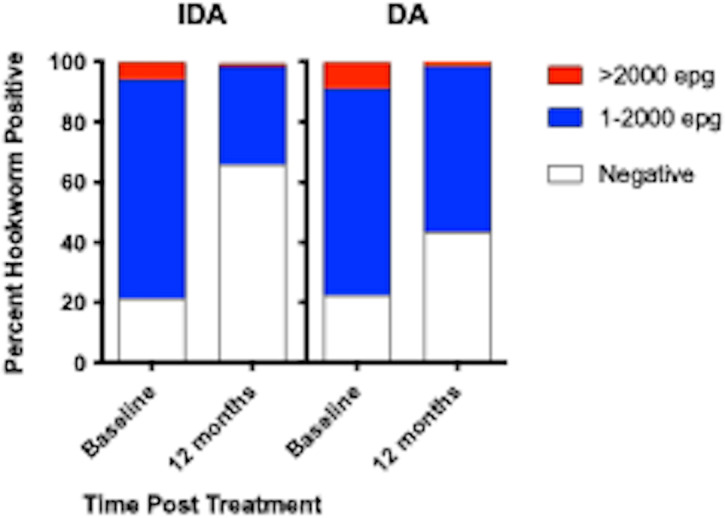
The impact of MDA on hookworm prevalence and intensity of infection stratified by treatment with IDA or DA. Hookworm prevalence was similar across age groups in participants in both treatment arms. IDA significantly reduced hookworm prevalence more than DA in all age groups (Fig 3).

At 12 months after MDA, the overall community prevalence of hookworm in the IDA arm was significantly lower, 34% (95%CI [29,40%]) compared to 57% (95%CI [51,62%]) in DA communities (*p*<0.001) (Table 2B and Fig 2).

**Fig 3:**
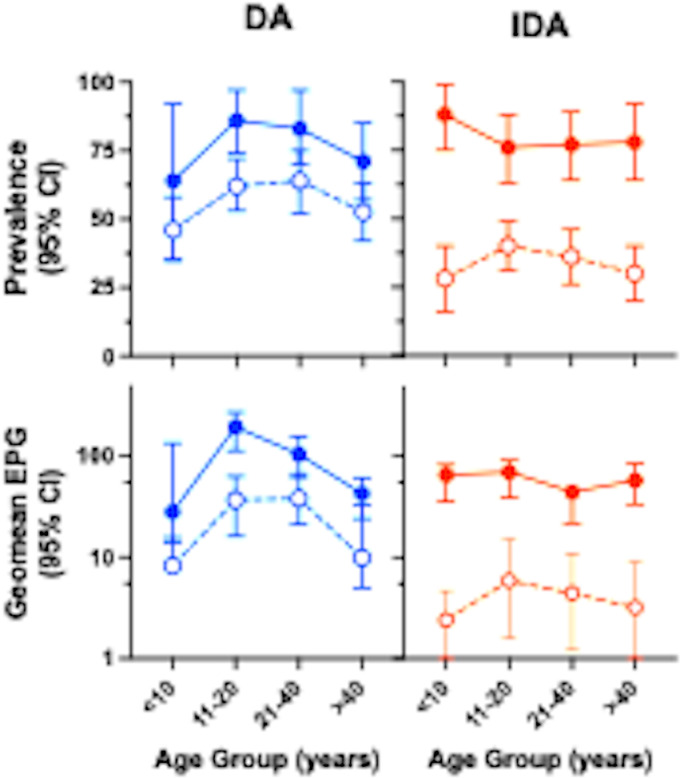
Hookworm prevalence (upper panel) and intensity (lower panel) by age group and treatment arm at baseline (solid circles) and 12 months post-MDA (clear circles with dashed lines). Blue are DA-treated communities, and red IDA treated communities. Prevalence or geomean epg and 95% Confidence intervals are shown. (S2 Table for sample size stratified by age).

IDA reduced the intensity of hookworm infection by 93% (geomean = 58 epg at baseline and geomean of 4 epg at 12 months; *p* < 0.001, Table 2A) compared to an 85% reduction in the intensity of infection for DA-treated villages (geomean=81 epg vs 12 epg; *p* < 0.001, Table 2B**).** This more significant reduction in the burden of hookworm infection in IDA versus DA-treated communities was driven by the more significant reduction in hookworm prevalence observed in IDA versus DA communities since there was no significant difference in geometric mean egg counts among individuals that were egg-positive at baseline and egg positive at 12 months after treatment (S3 Table). Many individuals who remained egg-positive at 12 months had not participated in MDA.

To account for possible differences in gender, age, and village coverage and baseline infection on treatment outcomes for all participants, we examined the odds of predicting hookworm infection using a logistic mixed model between participants living in IDA and DA villages (Table 3). IDA village residents were 45% less likely to have hookworm infections 12 months after treatment than residents of DA villages (*p* = 0.049).

**Table 3:** Model-adjusted odds ratio of predicting hookworm infection, comparing treatment arms at baseline and 12 months.

Odds Ratio Label	OR (95% CI)	*p*-value
IDA vs. DA Baseline	1 (0.44, 2.24)	0.997
IDA vs. DA, 12 months	0.55 (0.31, 1)	0.049

*P*-values were determined using a linear mixed model with PROC MIXED in SAS 9.4

### Individual versus community effects of MDA on hookworm infection

**Fig 4:**
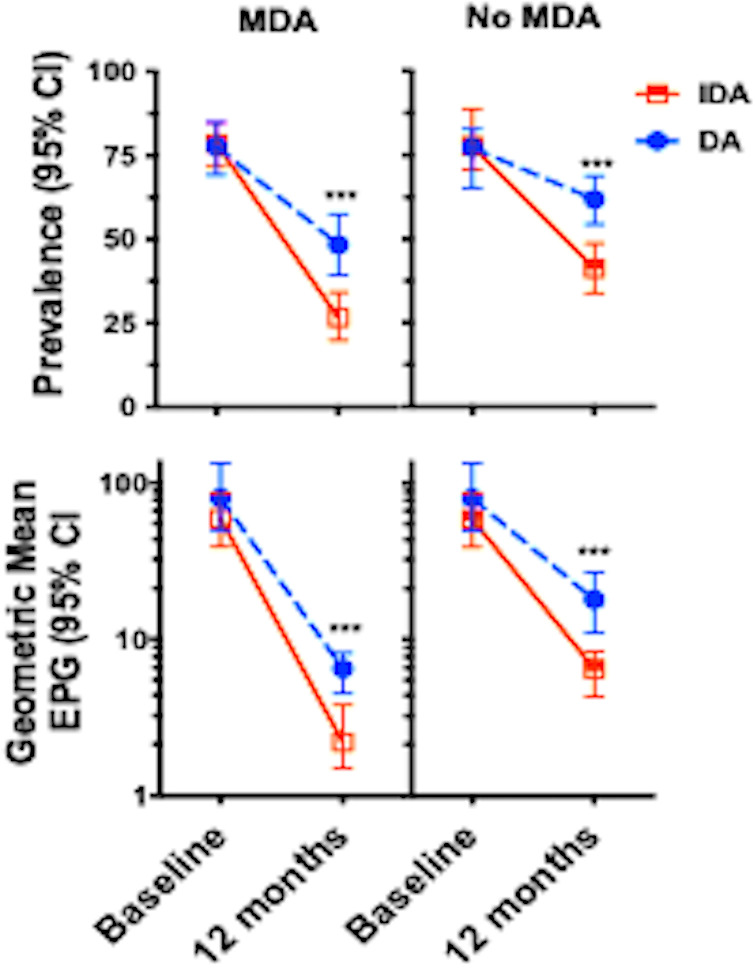
Hookworm prevalence and intensity 12 months after MDA among individuals who received treatment (N = 124 in DA and N = 158 IDA villages) or those who did not (N = 186 in DA and N = 182 in IDA villages). Values are mean prevalence (95% CI) or geometric mean epg (95% CI). *** *p* < 0.001.

To examine the indirect effects of MDA on hookworm infection at 12 months after treatment, we compared the prevalence and intensity of hookworm infection in a group of individuals who received MDA versus those who did not receive or could not verify MDA treatment (Fig 4).

Among individuals who received MDA with DA, there was a 39% reduction in hookworm prevalence (78% to 48% *p* < 0.001) compared to a 66% reduction in prevalence among those who had received IDA (80% to  27%, *p* < 0.001, Fig 4 upper left panel). The greater percent reduction in IDA-treated individuals was highly significant (*p* =< 0.001, chi-square). The reduced prevalence of hookworm among individuals residing in the same communities who had not participated in MDA was notable. There was a 21% decrease in hookworm prevalence (79 to 62%, *p* =< 0.001) in DA-treated villages and a 47% reduction in prevalence (80% to 41% *p* < 0.001, Fig 4 upper right panel) in IDA-treated communities. This reduction in prevalence following MDA was significantly greater in the IDA-treated communities compared to DA treated villages (*p* =< 0.001, chi-square). A similar pattern of reduction in the hookworm intensity of infection occurred in the same groups of individuals stratified by treatment group and whether they received treatment (Fig 4 lower panels).

### *Strongyloides* infection

Stools were examined by PCR for *Strongyloides spp* before and after MDA. Only 28 stool samples were PCR-positive, which is too small of a sample size for any meaningful analysis. All samples were *S. stercoralis* based on species-specific primers and sequencing of the amplicons. Therefore, we assessed the presence of *Strongyloides* infection by antibodies reactive with the recombinant *S. stercoralis* antigen NIE.

**Fig 5:**
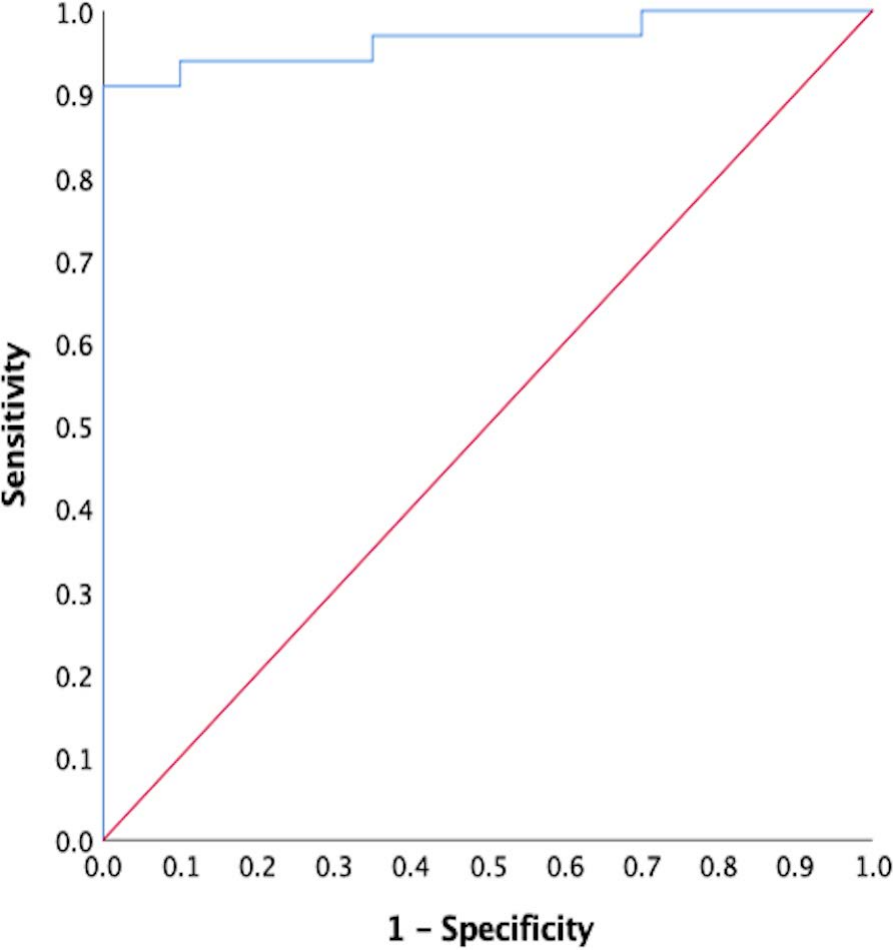
Receiver operating characteristic curve for anti-NIE antibodies for *S. stercoralis.* Using this cut-off, 192 of 283 (68%) in the IDA arm and 180 of 290 (62%) in the DA arm were antibody positive for the *S. stercoralis* NIE antigen at baseline. Twelve months following MDA, the seroprevalence decreased in the IDA arm to 88 of 212 (41.5%) compared to 100 of 185 (54%) in the DA arm (*p* = 0.012, chi-square) (Table 4A). The seroprevalence increased with age and was significantly decreased following IDA treatment in each age group (Fig 6). The seroprevalence did not significantly decrease following DA treatment. These results include all individuals sampled, irrespective of whether they took the medications. For participants with paired samples, baseline *S. stercoralis* prevalence in the IDA arm was 144 of 209 (69%) and 95 of 179 (53%) in the DA arm with slightly higher baseline OD levels (Table 4B and Fig 7). At 12 months following treatment, 22 individuals seroreverted to negative in the DA arm, whereas 71 seroreverted in the IDA arm (to 49%) (Table 4B). There was a reduction in mean OD (*p* < 0.0001) in paired samples from participants in the IDA arm and no reduction in mean OD for the DA arm (Fig 7). Twenty-one samples that initially were negative became positive at 12 months in the DA arm, whereas 14 became positive in the IDA arm.

Nine hundred seventy dried blood spots were evaluated from 582 participants, 573 at baseline and 397 at 12 months for antibodies reactive to NIE. Of the total samples, 388 were paired at baseline and 12 months post-MDA. Receiver operating characteristic curve (ROC) analysis of PCR confirmed positives for *Strongyloides* (*n* = 33), and North American negative controls (*n* = 20) assigned ELISA cut-off values. At a cut-off of 0.067 optical density (OD) units, the NIE ELISA sensitivity was 91%, and the specificity was 90% (ROC area under the curve=0.96) (Fig 5).

**Table 4:** *Strongyloides* antibody seroprevalence at the community level (A) and paired samples (B).

Panel A								
Community			Baseline			12 months		
Treatment Arm	Median Age, Years (range)	Gender Male %	*n*	Prevalence %		*n*		Prevalence %
**IDA**	28 (5–78)	59	283	192 (68%)		212		88 (41.5%)
**DA**	30 (5–75)	59	290	180 (62%)		185		100 (54%)
*P*-value	0.5	>0.99		0.132				0.012
**Panel B**								
**Paired Samples**					**Baseline**		**12 months**	
**Treatment Arm**	*n*	**Median Age, Years (range)**	**Gender Male %**		**No. Pos.** **Prevalence %**		**No. Pos.** **Prevalence %**	
IDA	209	29 (6–78)	61		144 (69%)		85 (41%)	
DA	179	33 (5–75)	59		95 (53%)		94 (52.5%)	
*P*-value		0.249	0.678		0.001		0.023	

**Fig 6:**
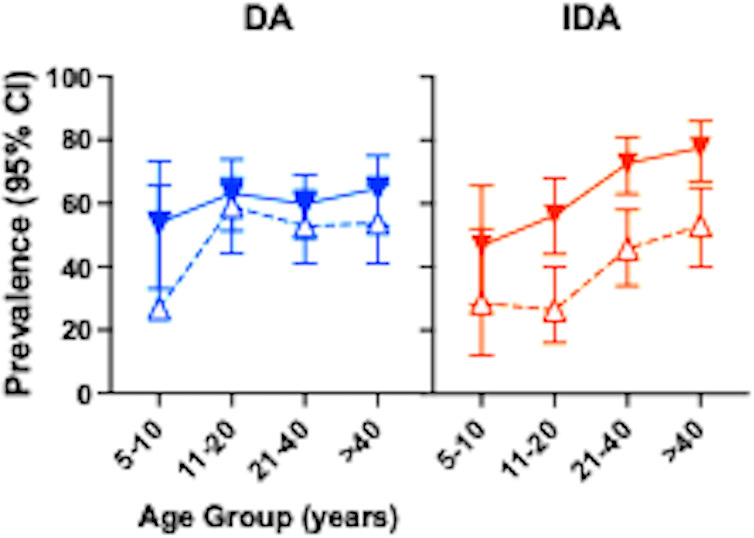
The impact of MDA with DA or IDA on seroprevalence of *Strongyloides* stratified by age. Solid triangles indicate prevalence at baseline (95% CI) and open triangles (95% CI) 12 months following MDA.

**Fig 7:**
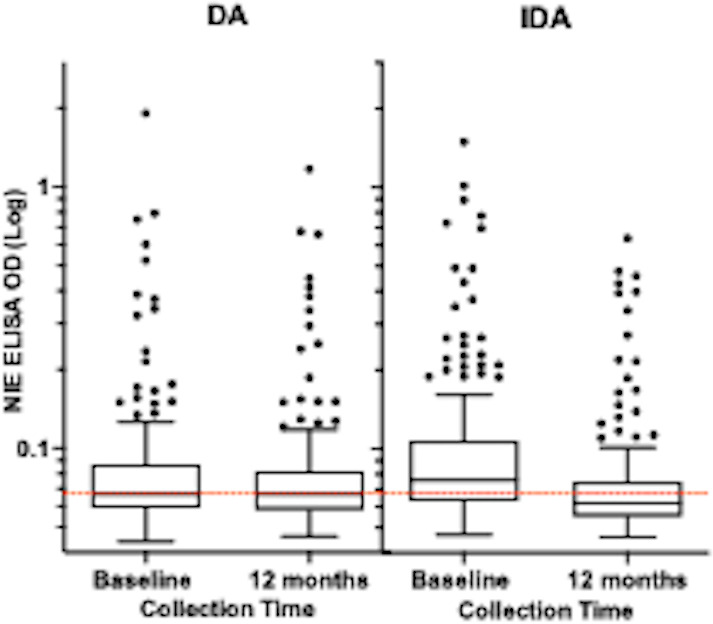
Changes post-treatment in NIE ELISA values in the IDA arm vs. DA arm. Data are from paired samples shown in Table 4B. The dashed line represents the threshold for cut-off for a positive sample based on the receiver operating characteristic curve shown in Fig 5. Mean ODs are shown in red lines. **** P < 0.0001 (Wilcoxon matched pairs sign ranked test).

### NIE antibody reactivity and lymphatic filariasis and hookworm infection

A subgroup of 265 NIE ELISAs from participants who provided stool samples at baseline was analyzed to see if NIE seropositivity was associated with LF and hookworm infection suggestive of cross-reactivity. From 101 NIE ELISA-positive samples, 22 were circulating filarial antigen (CFA) positive, and 80 were positive for hookworm by Kato Katz. A Mann Whitney U test showed no significant difference between NIE ELISA OD and LF status by FTS test result (*p*=0.366), and NIE ELISA OD and hookworm status by Kato Katz results (*p*=0.482).

## Discussion

This study found that a single round of MDA with IDA or DA markedly reduced the *community* burden of hookworm infection one year after treatment in communities with high baseline hookworm prevalence. IDA reduced community prevalence by 63% compared to 43% after DA based on hookworm ova in stool using Kato Katz. Both treatment regimens significantly reduced the proportion of individuals with >2000 epg at baseline (moderate and heavy infections as classified by WHO) by 82.4% for IDA and 82.8% for DA 12 months later. However, this reduction was comparable between treatment groups. Using a multivariate model adjusting for age, sex, drug coverage in the different villages, and baseline infection levels, communities treated with IDA showed a 45% greater reduction in hookworm prevalence compared to communities treated with DA, suggesting a beneficial effect of the triple-drug combination beyond LF alone in populations with coendemic hookworm infections. IDA treatment did not reduce heavy infection (>2000 egg/gm) any better than DA but appears more effective in those with lower intensity of infection. Notable was a significant indirect effect of MDA on hookworm infection because untreated individuals also showed a decline in the prevalence and intensity of infection 12 months after MDA was implemented. This indirect effect was more pronounced in communities receiving IDA than DA. Thus, MDA reduced hookworm transmission.

Clinical trials of treatments for hookworm have shown only a marginal benefit in drug efficacy for IVM plus ALB combination therapy over ALB alone [19,37]. The impact of MDA with DEC plus ALB in areas outside sub-Saharan Africa or with IVM plus ALB or ALB alone in various LF endemic areas in sub-Saharan Africa has shown a significant impact on the community burden of STH infection with one or more annual rounds of MDA [17,38,39]. However, directly comparing these studies is difficult because baseline STH prevalence, MDA coverage, local environmental conditions, and hookworm species vary greatly among study sites. Only *N. americanus* was observed in this PNG study, whereas *A. duodenale* also occurs in West Africa and Indonesia, where IVM plus ALB has been compared to ALB alone [17,38,39]. This is the first study to examine the relative benefits of MDA with IDA versus DA on hookworm and strongyloidiasis infections in a cluster randomized trial. This difference was observed across different age groups and remained significant when adjusted for location and coverage. At 12 months, individuals who provided stool samples were similar regarding age, sex, and the number of individuals treated at baseline between the two treatment arms.

*Strongyloides* infections were *S. stercoralis* based on stool samples detected as PCR positive. Tests for antibodies to the *S. stercoralis* NIE antigen which is present in infective L3 larvae [36], have improved the diagnosis of *S. stercoralis* infection. Such antibodies indicate recent or active infection [40] with high sensitivity and specificity [6]. The lack of an association with antibodies to NIE and hookworm or LF infection in the current study supports the specificity of this assay [41]. As expected, villages treated with DA showed no overall change in the prevalence of *S. stercoralis* by serology or in antibody levels among individuals that did not serorevert. In contrast, MDA with IDA led to a 39% reduction in antibody prevalence, and antibody levels also decreased in individuals who did not fully serorevert. These results add to prior observations on the impact of MDA with IVM on *S. stercoralis* prevalence. In the Northern Territory of Australia, a single round of IVM MDA reduced *S. stercoralis* seroprevalence from 21% at baseline to 6% at 12 months, with a further reduction to 2% after a second round of MDA [42]. Multiple rounds of MDA with IVM in Ecuador reduced the seroprevalence from 6.8% to zero [43]. In the Solomon Islands, one round of MDA with IVM for scabies in children 0-12 years of age decreased *Strongyloides* seroprevalence from 9.3% to 5.1% by 12 months after treatment [44].

Few studies have investigated *S. stercoralis* prevalence in PNG. A study of anemia in pregnant women in the highland area of Goroka found a prevalence of 3% by a direct wet mount with saline [45] and a second study of children in Morobe province reported a prevalence between 4-12% by Harada-Mori culture [2]. In addition to *S. stercoralis*, another species of *Strongyloides* found in humans in PNG, *Strongyloides fuelleborni kellyi*, reported in the 1970s by Kelly et al. [31], affecting mainly infants and causing a fatal disease known locally as Swollen Belly Syndrome, with an estimate prevalence between 70-100% in children under five years of age living in high prevalence areas [3,4,32]. PCR-positive amplicons were sequenced in this study, and all were *S. stercoralis*.

Remarkably, we found no *Trichuris* parasites and only two samples with *Acaris* infection by Kato Katz detection. This lack of STH species transmitted by fecal-oral route and the high prevalence skin-penetrating STH species is unusual. We observed that residents in this area of PNG are careful about personal hygiene, which may account for the lack of fecal-oral STH species. Area residents establish bush pit toilets and use them routinely rather than defecating anywhere. We speculate that these bush toilets provide ideal sites for the growth and amplification of the skin-penetrating larvae of hookworm and *Strongyloides*. Therefore, a key element of STH control in this area would be establishing improved bush toilets that reduce the risk of skin-penetrating STHs.

The current study’s limitations were that stool samples were not randomly obtained from village members; only 5–6% of individuals provided stool samples at baseline, and 14% of participants one year later of the total population in the study communities. Therefore, accurate estimates of population prevalence are compromised. However, this bias may have less impact on comparing MDA with IDA or DA. The villages were randomly assigned treatment arms. Stool sample collections varied among villages. Residents from one village refused to provide stools samples. However, there was no evidence that villages in one treatment arm were more likely to provide stool samples than those in the other treatment arm. This is highlighted in that the number of stool donors in the two MDA areas was similar and were of comparable age and sex. This does not exclude the possibility of other undetectable biases. Another limitation was that baseline stool samples were obtained from 12 clusters equally divided by IDA and DA treatment arms, whereas 12-month post-treatment samples were obtained from 23 villages. We believe smaller baseline sample numbers are unlikely to have biased outcomes, because hookworm infection was similar across villages before MDA, and the multivariant analysis adjusted for village coverage and baseline infection status. We plan to follow up with the communities to assess whether transmission interruption was sustained for LF, which includes a collection of DBS. This will allow us to examine changes in *Strongyloides* prevalence as determined anti-NIE antibodies in IDA-treated compared to DA-treated villages.

This study highlights the added benefit of MDA with IDA that now includes ivermectin that treats non-target parasites like *Strongyloides*, and other STHs that are highly prevalent in PNG. The treatment of adults with the highest-burden skin-penetrating STH is essential in reducing STH transmission in communities, especially among children and reproductive-aged women, a concept underlying the large multinational DeWorm3 clinical trial [46,47]. Whether MDA for STH can interrupt STH transmission in some areas is still under assessment in the DeWorm3 trial. In countries like PNG, with a high prevalence of hookworm and *Strongyloides*, MDA with IDA for LF combined with improved toilets and sanitation facilities could have a large impact on the STH burden.

## Supporting information

S1 Fig Hookworm cure rates at 12 months post-MDA for the cohort who received MDA at baseline. (DOCX)

S1 Table Hookworm prevalence and intensity of infection by Kato Katz at 12 months post-MDA where individuals were evaluated both at baseline and 12 months later. (DOCX)

S2 Table The sample size for age-stratified data. (DOCX)

S3 Table Model-adjusted geometric mean hookworm ova/gm (95% CI) among individuals with any hookworm infection. Using the linear mixed model with PROC MIXED in SAS 9.4 *p*=0.308 for IDA versus DA at 12 months.
(DOCX)

S1 Data NIE ELISA results. (XLSX)

S2 Data Kato Katz and PCR database 12 months collection. (XLSX)

S3 Data Master database baseline, 4 weeks and 1 year. (XLSX)
