# Supplementary figures and images for: Impact of mass drug administration with ivermectin, diethylcarbamazine, and albendazole for lymphatic filariasis on hookworm and Strongyloides stercoralis infections in Papua New Guinea
Source: PLoS Negl Trop Dis. 2025 Mar 10;19(3):e0012851. doi: 10.1371/journal.pntd.0012851 (PMC11893124; doi:10.1371/journal.pntd.0012851)

**S1 Fig.** Hookworm cure rates at 12 months post-MDA for the cohort who received MDA at baseline.


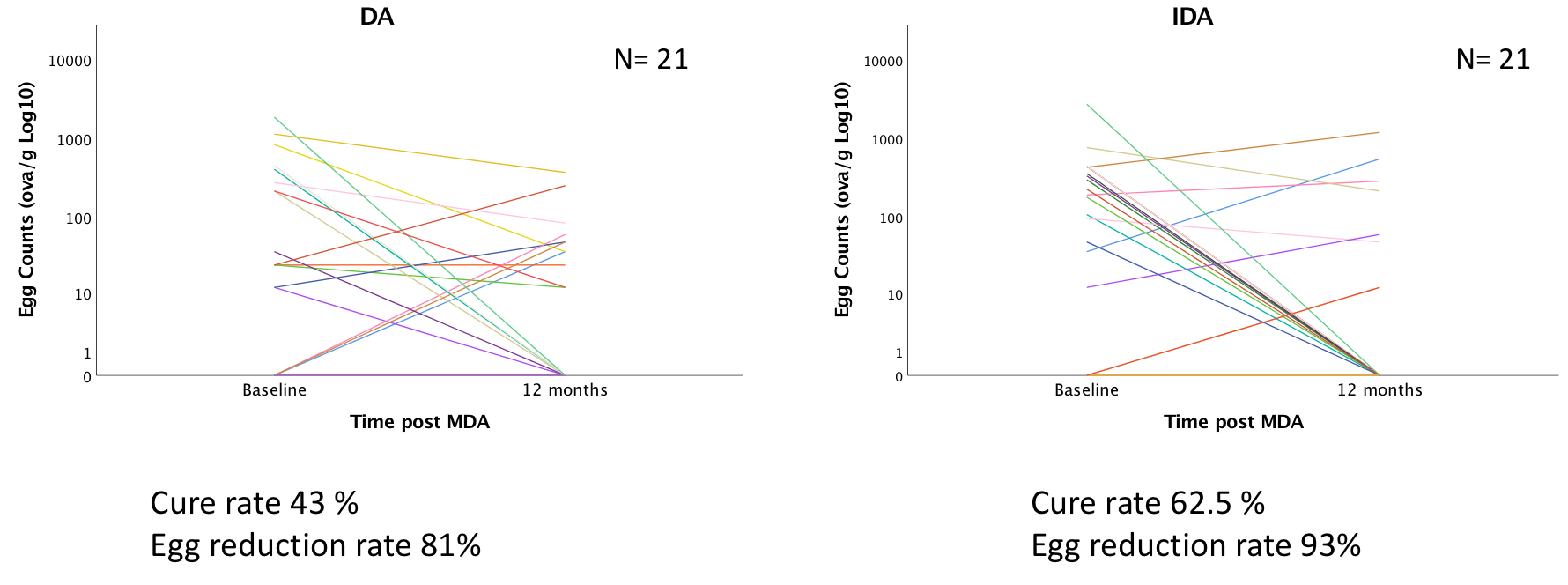

Supplement: S1 Fig — (DOCX) [file pntd.0012851.s001.docx]
