# Supplementary material for: Impact of mass drug administration with ivermectin, diethylcarbamazine, and albendazole for lymphatic filariasis on hookworm and Strongyloides stercoralis infections in Papua New Guinea
Source: PLoS Negl Trop Dis. 2025 Mar 10;19(3):e0012851. doi: 10.1371/journal.pntd.0012851 (PMC11893124; doi:10.1371/journal.pntd.0012851)
Supplement: S1 Table — (DOCX) [file pntd.0012851.s002.docx]

**S1 Table.** Hookworm prevalence and intensity of infection by Kato Katz at 12 months post-MDA where individuals were evaluated both at baseline and 12 months later.

| **Treatment Arm** | ***n*** | **Median Age, Years (range)** | **Gender Male%** | **Prevalence %** | **Geomean (95% CI) ova/g** | **Mean % Egg Reduction* (95% CI)** |
| --- | --- | --- | --- | --- | --- | --- |
| **DA** | 21 | 33 (6-60) | 67 | 52 | 6.8 (2.9,21) | 81 (61,101) |
| **IDA** | 21 | 16 (5 -57) | 67 | 38 | 4.9 (1.8,19) | 93 (83,103) |
| ***P-*value** |  | 0.12 | 1 | 0.35 | 0.60 | 0.17 |

*Egg reduction rate relative to baseline (pre-treatment)
