# Supplementary material for: Impact of mass drug administration with ivermectin, diethylcarbamazine, and albendazole for lymphatic filariasis on hookworm and Strongyloides stercoralis infections in Papua New Guinea
Source: PLoS Negl Trop Dis. 2025 Mar 10;19(3):e0012851. doi: 10.1371/journal.pntd.0012851 (PMC11893124; doi:10.1371/journal.pntd.0012851)
Supplement: S2 Table — (DOCX) [file pntd.0012851.s003.docx]

**S2 Table.** The sample size for age-stratified data

| **Treatment arm** | **<10 years** | **11-20 years** | **21-40 years** | **>40 years** |
| --- | --- | --- | --- | --- |
| **DA (baseline)** | 11 | 35 | 30 | 41 |
| **DA (12 months)** | 71 | 93 | 66 | 80 |
| **IDA (baseline)** | 25 | 45 | 47 | 32 |
| **IDA (12 months)** | 57 | 112 | 91 | 80 |
