# Supplementary material for: Impact of mass drug administration with ivermectin, diethylcarbamazine, and albendazole for lymphatic filariasis on hookworm and Strongyloides stercoralis infections in Papua New Guinea
Source: PLoS Negl Trop Dis. 2025 Mar 10;19(3):e0012851. doi: 10.1371/journal.pntd.0012851 (PMC11893124; doi:10.1371/journal.pntd.0012851)
Supplement: S3 Table — Using the linear mixed model with PROC MIXED in SAS 9.4 p=0.308 for IDA versus DA at 12 months. (DOCX) [file pntd.0012851.s004.docx]

**S3 Table**. Model-adjusted geometric mean hookworm ova/gm (95% CI) among individuals with any hookworm infection.

| **MDA Arm** | **Baseline Ova/gm**  **(95% CI)** | **12 months Ova/gm (95% CI)** |
| --- | --- | --- |
| DA | 343 (227,518) | 94 (68,129) |
| IDA | 207 (140,308) | 114 (82,160) |

Using the linear mixed model with PROC MIXED in SAS 9.4 *p*=0.308 for IDA versus DA at 12 months.
